# Fitness Levels and Gender Are Related With the Response of Plasma Adipokines and Inflammatory Cytokines in Prepubertal Children

**DOI:** 10.3389/fnut.2022.883871

**Published:** 2022-04-21

**Authors:** Francisco Jesus Llorente-Cantarero, Concepción M. Aguilera, Juan Luis Perez-Navero, Angel Gil, Juan de Dios Benitez-Sillero, Mercedes Gil-Campos

**Affiliations:** ^1^Department of Specific Didactics, Faculty of Education, Maimónides Institute of Biomedicine Research of Córdoba (IMIBIC), University of Córdoba, Córdoba, Spain; ^2^Center of Biomedical Research on Physiopathology of Obesity and Nutrition (CIBEROBN), Institute of Health Carlos III (ISCIII), Madrid, Spain; ^3^Department of Biochemistry and Molecular Biology II, Institute of Nutrition and Food Technology “José Mataix”, Center of Biomedical Research, University of Granada, Granada, Spain; ^4^Instituto de Investigación Biosanitaria. ibs, Granada, Spain; ^5^Metabolism and Investigation Unit, Reina Sofia University Hospital, Maimónides Institute of Biomedicine Research of Córdoba (IMIBIC), University of Córdoba, Córdoba, Spain

**Keywords:** inflammation, adipokines, fitness, exercise, childhood, cytokines

## Abstract

**Background and Aim:**

Changes in adipokines have been related with the development of metabolic syndrome, frequently associated with obesity, and other risk factors. Fitness seems to promote a healthy cardiovascular status and could be a protector factor, just from childhood. Therefore, the present study aimed to evaluate the relationship between fitness levels with plasma adipokines and inflammatory biomarkers in prepubertal children.

**Methods:**

One hundred and thirty-seven healthy normal-weight prepubertal children were recruited from local schools and divided after performing the fitness tests, into two groups according to fitness level—low cardiovascular fitness group (LF) and equal or higher cardiovascular fitness group (HF). Anthropometric variables, blood pressure (BP) and plasma insulin, and leptin, resistin, adiponectin, tumor necrosis factor-alpha, hepatic growth factor, interleukin (IL)-8, monocyte chemoattractant protein-1, nerve growth factor (NGF), and plasminogen activator inhibitor-1 (PAI-1) were measured fasting in both groups to be compared. Univariate analysis of variance, comparative analysis, binary logistic regression, stepwise linear regression, and principal component analysis were conducted to evaluate the association between fitness, BMI, gender, and the biochemical parameters.

**Results:**

Girls and boys with HF presented lower waist circumference Z-score, BMI Z-score, systolic BP (only boys) as well as lower levels of leptin and NGF compared with their respective LF group. Regarding the association between variables, fitness showed an inverse relationship with BMI Z-score, leptin, PAI-1, HOMA-IR, resistin, IL-8, and NGF.

**Conclusion:**

An adequate level of fitness seems to protect against risk factors related to low-grade inflammation and altered adipokines that are related to the onset of obesity just from the prepubertal stage.

## Introduction

Currently, it is known that adipocytes secrete a variety of proteins called adipokines that are involved in different biological functions (1). Proinflammatory cytokines—such as interleukin (IL)-8, tumor necrosis factor-alpha (TNF-α) and other proteins as C-reactive protein (CRP)—are increased in cardiovascular disease (CVD), type II diabetes and obesity (1). Moreover, several adipokines such as leptin and adiponectin play a role in glucose and lipid metabolism, and energy homeostasis, and alterations have been reported in them, related to a higher risk for the development of these pathologies, already in prepubertal children (2). To evaluate the significance of cytokines modulation in pathological conditions, it is necessary to establish the physiological ranges of these molecules in healthy subjects, mainly from early childhood; considering that obesity and metabolic syndrome risk factors are present in scholars (3). In addition, it should be considered that children are in continuous growth and will present changes at different stages of puberty; therefore it is important to study the low inflammatory status response at these early stages to evaluate the influence of all these factors (4).

On the other hand, low fitness levels have been associated with being more prone to develop metabolic risk, being able to act through different pathways, including changes in inflammatory status (5, 6). Infact, an adequate fitness level has been negatively associated with cardiovascular mortality (7), acting as a protector factor relatively static (7). During childhood, cardiorespiratory fitness (CRF) has been inversely correlated with traditional CVD risk factors (6) and low-grade inflammation (8). One of the mechanisms by which physical fitness might promote cardiovascular health is by supporting anti-inflammatory processes.

Moreover, differences between boys and girls have been observed mainly in those biomarkers related to adipose tissue metabolism (1). Although causality it is not well-established, some hypotheses attribute these differences to puberty changes (9). However, there is scarce knowledge about these differences and changes at the prepubertal stage.

As further research on boys and girls is necessary to clarify the role of fitness levels in adiposity, low-grade inflammation or other CVD risk factors (8, 10), this study aimed to evaluate the relationship between CRF levels, measured by alpha fitness battery (11), and adipokines and inflammatory biomarkers in healthy prepubertal boys and girls to provide more specific knowledge of the influence that both gender and fitness can have on a low state of inflammation and on the health status already in prepubertal children.

## Materials and Methods

### Subjects

Healthy prepubertal children were recruited from different local elementary schools in Córdoba, Spain. Inclusion criteria were: prepubertal stage (Tanner I) and age between 7 and 12 years). Exclusion criteria were: pubertal stage, disease, a long period of rest due to illness, and the use of any medication or diet that alters blood pressure or metabolism.

### Clinical Examination

A group of pediatricians examined children's medical histories and performed a physical examination to exclude any illness. Sexual maturity was assessed by physical examination using the Tanner five-stage scale. Weight, height, and waist circumference (WC) were measured using standardized techniques. Body mass index (BMI) was calculated as weight (kg)/height (m^2^). The values obtained for these anthropometric measurements were compared with the Spanish references (12). Systolic and diastolic blood pressure (BP) were measured using a random-zero sphygmomanometer (Dinamap V-100).

### Fitness Evaluation

Children were asked to perform the 20 m shuttle run test (20-mSRT) to evaluate their fitness level using a validated scale from Léger et al. (13). Subjects started running at an initial speed of 8 km/h, and the sprint was increased by 0.5 km/h at 1 min intervals (1 min = one stage), reaching 18.0 km/h by minute 20. Running speed cues were indicated by signals emitted by a commercially-available CD–ROM, and it is included in the ALPHA health-related fitness test battery (11).

Fitness level was considered to divide the sample in two groups. The value obtained after the 20-mSRT was the criteria to include the participants into one or another group: children with a score equaling or above the average of the reference values (14) were assigned to the group designated as “equal or higher cardiovascular fitness group” (HF), and those with a score under the average were assigned to the “low cardiovascular fitness” group (LF).

### Cytokines Analysis

Blood samples were obtained from all the children using an indwelling venous line to draw a 3 ml sample after a 12-h overnight fasting. After centrifugation, aliquots of plasma were frozen immediately and stored at −80° until analyzed. C-reactive protein (CRP) was determined using a high-sensitivity, particle-enhanced turbidimetric immunoassay (PETIA) (Dade Behring Inc., IL). LINCOplex^TM^ kits of human monoclonal antibodies (Linco Research, MO, USA) were analyzed on a Luminex® 200™ System (Luminex Corporation, Austin, TX, USA) to determine: adiponectin (CV: 9.2%) (Cat. #HCVD1-67AK), resistin (CV: 6.0%) (Cat. HADK1-61K-A), leptin (CV: 7.9%) (Cat. #HADK2-61K-B), plasma hepatic growth factor (HGF) (CV: 7.7%), interleukin (IL)-6 (CV: 7.8%), IL-8 (CV: 7.9%), monocyte chemoattractant protein-1 (MCP-1) (CV: 7.9%), nerve growth factor (NGF) (CV: 6%), plasminogen activator inhibitor-1 (PAI-1) (CV: 11.8%), and TNF-α (CV: 7.8%) levels, according to manufacturer's instructions (15).

### Statistical Analysis

Data are expressed as mean ± SD. Normal data distribution was assessed by the Kolmogorov–Smirnov test. Homogeneity of variances was estimated using the Levene test. Inflammation data were log-transformed. Univariate Analysis of Variance was used to evaluate the effect of fitness and gender on cytokines and inflammatory biomarkers adjusted for age and BMI Z-score. Comparative analysis of two independent samples grouping by their levels of fitness or gender was performed using the Mann–Whitney test. General statistics analysis, binary logistic regression and stepwise linear regression were conducted to evaluate the association between fitness, gender and inflammation biomarkers. Principal component analysis (PCA) was performed to investigate the relationships among body mass index, peripheral tissue insulin resistance—as a risk feature of metabolic syndrome—fitness levels, and adipokines and inflammatory biomarkers in the 137 children. Extraction of the initial set of uncorrelated components was accomplished with the principal factor method, and then Varimax orthogonal rotation of components was used to facilitate interpretation. High loading values indicate a stronger relationship between a factor and an observed variable. Factor loadings lower than 0.359 (critical factor, *p* < 0.001) revealed marginal correlations. All statistical procedures were conducted using SPSS (IBM SPSS Statistics, Version 25.0. Armonk, NY, USA).

## Results

### General Comparisons

A total of 137 children were recruited: 72 were included in the HF group and 65 in the LF group. Table 1 shows the demographic and anthropometric characteristics and BP levels of this sample, as well as plasma cytokines, divided by gender and by fitness levels. No differences in age and BP were observed between groups classified by fitness. BMI was higher in the LF group. By gender, girls were younger and presented significantly lower BMI, SBP, and DBP values than boys. By level of fitness, girls and boys with HF (GHF and BHF, respectively) showed lower WC Z-score, BMI Z-score, and SBP (only boys) than girls and boys with LF (GLF and BLF, respectively). In addition, GLF presented lower values on all anthropometric variables than BLF. Lower WC, BMI, and SBP were observed in GHF compared with BHF.

**Table 1:** Anthropometric and demographic variables as well as concentration of plasma adipokines and inflammatory cytokines classified by grouped by fitness levels and gender.

	**LF**	**HF**	**Girls**	**Boys**	**G-LF**	**G-HF**	**B-LF**	**B-HF**
	**N: 65**	**N: 72**	**N: 53**	**N: 84**	**N: 32**	**N: 21**	**N: 33**	**N: 51**
Age (years)	9.67 ± 1.09	9.58 ± 1.22^**§**^	8.79 ± 0.88	10.18 ± 0.98^§^	9.00 ± 0.67	8.48 ± 1.08	10.35 ± 1.02	10.04 ± 0.95
WC (cm)	70.97 ± 11.66	63.04 ± 7.56	63.33 ± 9.08	69.62 ± 11.20^§^	65.60 ± 10.25^**§**^	59.88 ± 5.56^*^	76.70 ± 10.40^**§**^	64.34 ± 7.93^*^
Z-score WC	0.98 ± 1.42	−0.10 ± 0.96^**§**^	0.36 ± 1.32	0.51 ± 1.36	0.59 ± 1.47^*^	0.01 ± 0.98	1.39 ± 1.26^**§**^	−0.14 ± 0.97
BMI (kg/m^2^)	21.49 ± 4.04	18.62 ± 2.84^**§**^	18.98 ± 3.69	20.84 ± 3.80^+^	19.93 ± 17.52^**§**^	17.51 ± 2.53^+^	23.09 ± 3.42^**§**^	19.08 ± 2.87^*^
Z-score BMI	1.19 ± 1.20	0.32 ± 0.90^**§**^	0.47 ± 1.08	0.96 ± 1.19^*^	0.71 ± 1.17^**§**^	0.11 ± 0.81^*^	1.67 ± 1.05^**§**^	0.41 ± 0.93
SBP (mmHg)	123 ± 16.22	120.06 ± 10.49	115.19 ± 13.40	126.20 ± 13.08^§^	114.53 ± 15.57^**§**^	116.19 ± 9.45	131.74 ± 11.74^**§**^	121.68 ± 10.57^*^
DBP (mmHg)	67.14 ± 10.53	67 ± 9.26	63.77 ± 10.23	69.26 ± 9.12^§^	63.53 ± 64.14^+^	64.14 ± 8.03	70.87 ± 7.90	68.20 ± 9.55
Adiponectin (nmg/l)	20.45 ± 16.71	21.09 ± 17.55	26.30 ± 24.93	17.55 ± 12.46^*^	24.62 ± 22.95	28.74 ± 27.98	16.42 ± 4.03	17.90 ± 9.33^*^
PAI-1 (μg/l)	19.03 ± 10.56	13.93 ± 9.53^**§**^	15.28 ± 6.83	17.63 ± 12.46	16.58 ± 6.36	13.31 ± 7.20	21.56 ± 13.26	14.18 ± 10.34
Resistin (μg/l)	12.85 ± 4.66	12.95 ± 7.19	13.40 ± 5.85	12.56 ± 6.15^+^	13.06 ± 4.09^*^	13.93 ± 7.91	12.63 ± 5.24	12.54 ± 6.91
HGF (ng/l)	1861.35 ± 6115.71	1887.97 ± 919.99	1821.65 ± 703.67	1925.63 ± 829.72	1879.22 ± 502.19^**+**^	1733.94 ± 939.74	1842.91 ± 722.61	1951.39 ± 913.53
IL-8 (ng/l)	3.42 ± 2.77	3.92 ± 3.01	3.12 ± 0.91	4.00 ± 3.52	3.13 ± 0.85	3.09 ± 1.02	3.71 ± 3.87	4.26 ± 3.47
Leptin (ng/l)	21927.41 ± 18794.85	8925.58 ± 8148.46^*^	12729.94 ± 10151.91	17467.79 ± 18713.56^+^	15634.66 ± 10944.81^*^	8303.70 ± 6943.74	28423.14 ± 22812^*^	9186.78 ± 8656.69
MCP1 (ng/l)	147.50 ± 67.49	163.56 ± 96.86	154.42 ± 65.84	159.91 ± 98.59	153.93 ± 58.91	155.18 ± 76.74	140.87 ± 75.74	167.01 ± 104.52
NGF (ng/l)	11.22 ± 6.69	9.06 ± 5.80^*^	9.47 ± 7.60	10.26 ± 5.33^+^	11.28 ± 7.96	6.73 ± 6.25^**+**^	11.16 ± 5.19	10.01 ± 5.38^+^
TNF-α (ng/l)	7.20 ± 2.72	7.04 ± 2.85	6.28 ± 1.83	7.63 ± 3.11	6.55 ± 2.04	5.87 ± 1.39	7.86 ± 3.18	7.52 ± 3.16

*LF, low fitness group; HF, equal or higher fitness group; BMI, body mass index; SBP, systolic blood pressure; DBP, diastolic blood pressure; HGF: hepatocyte growth factor; IL-6, interleukin-6; IL-8, interleukin-8; MCP-1, monocyte chemoattractant protein-1; NGF, nerve growth factor; PAI 1, plasminogen activator inhibitor-1; TNF-α, tumour necrosis factor alpha*.

*Data are expressed as mean ± SD*.

§ *P < 0.001*;

+ *P < 0.010*;

* *P < 0.05*.

* *Statistical significance between groups of fitness (LF vs. HF) and between gender (girls vs. boys) after application of ANCOVA (analysis of covariance) adjusting by sex, age, WC, and BMI*.

*The comparison among groups is the following:*

*Second column: LF vs. HF; Fourth column: Girls (G) vs. Boys (B); Fifth column: G-LF vs. B-HF; Sixth column: G-LF vs. G-HF; Seventh column: B-LF vs. B-HF; Eighth column: G-HF vs. B-HF*.

Regarding the inflammation biomarkers, the LF group presented higher levels of leptin (*d* = 0.536) and NGF (*d* = 0.615) compared with the HF group. PAI-1 showed higher plasma levels in the LF group compared with the HF group (*p* = <0.001) before adjusting by BMI Z-score, but this significance disappeared after adjusting. When the fitness levels for boys and girls were considered, differences in adiponectin (*d*=0.506), resistin (*d* = 0.819), leptin (*d* = 0.460), HGF and NGF were observed. The GHF group presented the highest levels of anti-inflammatory parameters and the lowest in inflammatory markers after comparing with GLF, BHF, and BLF (Table 1).

### Principal Component Analysis

From the 12 items included in the PCA (fitness, body composition, insulin resistance, adipokines and inflammatory biomarkers), four principal components were extracted (Table 2), which explained 59.04% of the total variance (25% of the variance was explained by the first factor, an additional 13% by the second factor, another 11% by the third factor and other 8% by the fourth factor; Table 3). The first principal component, termed “Fitness” showed a positive correlation between HOMA-IR, leptin, BMI, PAI-1, and negative with resistin, and cardiorespiratory fitness. The second component, termed “Cytokines,” included correlations among PAI-1, IL-8, TNF- α, HGF and resistin. The third component named “Adiposity” included a positive correlation between BMI, Adiponectin, and MCP-1. The fourth component, termed “Growth Factors” included an inverse association between HGF and NGF.

**Table 2:** Principal component analysis for the study extracted from fitness, body composition, insulin resistance, adipokines, and inflammatory biomarkers variables.

	**Component matrix^a^**			
**Variables**	Factor^*^			
	**Fitness**		**Adiposity**	**Growth factors**
Leptin	0.777			
CF olds	−0.745			
HOMA-IR	0.703			
PAI 1	0.590	0.449		
BMI	0.417		0.399	
IL8		0.852		
TNF-α		0.809		
HGF		0.601		0.406
Resistin	0.334	0.360		
Adiponectin			0.764	
MCP-1			0.613	
NGF				−0.895

*CF olds, cardiorespiratory fitness classified by olds; HOMA-IR, homeostasis model assessment-insulin resistance; HGF, hepatocyte growth factor; IL-8: interleukin-8; MCP-1: monocyte chemoattractant protein-1; NGF: nerve growth factor; PAI 1: plasminogen activator inhibitor-1; TNF-α: tumour necrosis factor alpha*.

a *Extraction of the initial set of uncorrelated components was accomplished with the principal factor method, and then the Varimax orthogonal rotation of components was used to facilitate interpretation. The number of components retained was based on Scree plot analysis and eigenvalues >1 (with the components accounting for more of the total variance than any single variable)*.

* *Factor loading is the product-moment correlation (a measure of linear association) between an observed variable and an underlying factor. A significant loading factor was defined as a value >0.359 (p < 0.01)*.

**Table 3:** Eigen values and percentages of variance associated with each linear component (factor) before extraction, after extraction, and after rotation, in the principal component analysis for the study of children relating fitness, body composition, insulin resistance, adipokines, and inflammatory biomarkers variables.

	**Total variance explained**								
**Components**	**Initial eigenvalues**			**Sums of loads squared from extraction**			**Sums of loads squared of rotation**		
	**Total**	**% of variance**	**% accumulated**	**Total**	**% of variance**	**% accumulated**	**Total**	**% of variance**	**% accumulated**
1 (Fitness)	3.017	25.145	25.145	3.017	25.145	25.145	2.380	19.835	19.835
2 (Cytokines)	1.674	13.953	39.098	1.674	13.953	39.098	2.220	18.496	38.332
3 (Adiposity)	1.368	11.397	50.495	1.368	11.397	50.495	1.336	11.131	49.463
4 (Growth factors)	1.026	8.546	59.041	1.026	8.546	59.041	1.149	9.578	59.041

### Correlations and Regression

Table 4 shows significant correlations between cytokines, fitness and demographic variables. There was a strong positive correlation between WC Z-score and BMI Z-score with PAI-1 and leptin; and a negative correlation with adiponectin and fitness. Fitness was also negatively correlated with leptin. Regarding the relationship between the cytokines, it was observed a strong positive association between HGF, IL-8, leptin, and TNF- α.

**Table 4:** Significant correlations between adipokines, fitness, and demographic variables in healthy prepubertal children.

**Variables**	***R***	***P***	**Variables**	***R***	***P***
Age/Z-score BMI	0.228	0.007	Adiponectin/Sex	−0.295	0.001
Age/Z-escore WC	0.240	0.005	Adiponectin/Leptin	−0.168	0.050
Age/Adiponectin	−0.227	0.009	Adiponectin/MCP 1	0.222	0.010
Age/Leptin	0.170	0.047	Adiponectin/NGF	−0.176	0.044
Age/MCP 1	−0.217	0.012	Adiponectin/TNF-α	−0.186	0.033
Age/TNF-α	0.179	0.036	PAI 1/Resistin	0.277	0.001
Z-score WC/Adiponectin	−0.272	0.002	PAI 1/HGF	0.437	<0.001
Z-score WC/PAI 1	0.506	<0.001	PAI 1/IL 8	0.383	<0.001
Z-score WC/Resistin	0.257	0.002	PAI 1/Leptin	0.535	<0.001
Z-score WC/HGF	0.275	0.001	PAI 1/TNF-α	0.315	<0.001
Z-score WC/Leptin	0.779	<0.001	Resistin/HGF	0.253	0.003
Z-score WC/TNF-α	0.209	0.014	Resistin/Leptin	0.234	0.006
Z-score BMI/Adiponectin	−0.236	0.002	HGF/IL 8	0.422	<0.001
Z-score BMI/PAI 1	0.464	<0.001	HGF/Leptin	0.343	<0.001
Z-score BMI/Resistin	0.181	0.034	HGF/TNF-α	0.411	<0.001
Z-score BMI/HGF	0.238	0.005			
Z-score BMI/TNF-α	0.173	0.043	IL 8/MCP 1	0.240	0.004
CRF/Sex	0.221	0.010	TNF-α/Sex	0.226	0.007
CRF/Z-score WC	−0.411	<0.001	TNF-α/Resistin	0.192	0.023
CRF/Z-score BMI	−0.381	<0.001	TNF-α/IL 8	0.445	<0.001
CRF/PAI 1	−0.289	0.001	TNF- α/Leptin	0.239	0.005
CRF/Leptin	−0.419	<0.001	TNF-α/NGF	0.204	0.016
CRF/NGF	−0.172	0.045			

*BMI, body mass index; SBP, systolic blood pressure; DBP, diastolic blood pressure. HGF, hepatocyte growth factor; IL-8, interleukin-8; MCP-1, macrophage chemo attractant factor; PAI 1, plasminogen activator inhibitor-1; CRF, cardiorespiratory fitness; TNF-α, tumour necrosis factor alpha; r, Rho Spearman's correlation coefficient*.

After a multivariate logistic regression analysis (Table 5), gender showed associations with CRF, resistin, age, Z-score BMI, and TNF-α and an independent relationship with adiponectin. Moreover, fitness showed associations with BMI Z-score, NGF, and leptin.

**Table 5:** Logistic regression analysis for sex and fitness with different anthropometric variables and plams cytokines.

**Variables**	**Variables**	**B**	**Exp (B)**	***P***	**95% confidence interval of B**	
					**Lower bound**	**Upper bound**
**Sex**	Fitness	−2.378	0.093	<0.001	0.024	0.353
	Age	1.616	5.033	<0.001	2.586	9.797
	Z-score BMI	0.693	2.000	0.018	1.129	3.545
	Adiponectin	−0.028	0.973	0.199	0.933	1.015
	Resistin	−1.956	0.141	0.015	0.029	0.688
	IL-8	4.327	75.735	0.092	0.049	11694.26
	TNF- α	2.913	18.420	0.009	2.806	162.66
**Fitness**	Sex	−2.464	0.085	<0.001	0.024	0.300
	Age	−0.416	0.660	0.089	0.409	1.066
	Z-score BMI	−0.647	0.523	0.024	0.298	0.920
	Leptin	0.000	1.000	0.017	1.000	1.000
	NGF	−0.083	0.920	0.026	0.856	0.990
	IL-8	−2.555	0.078	0.098	0.004	1.602

*BMI, body mass index; IL-8, interleukin-8; NGF, nerve growth factor; TNF-α, tumor necrosis factor alpha*.

## Discussion

In the present study, changes in plasma cytokines were observed between prepubertal children with low or high fitness levels. In addition, adipokines and inflammatory biomarkers were affected by gender regardless of fitness levels, maybe due to a modulation of the adipose tissue on these biomarkers.

Preliminary evidences suggested that maintaining a healthy normo-weight during childhood and adolescence might be the most effective strategy to prevent chronic low-grade inflammation and cardiovascular and metabolic diseases in the future (16). It has also been demonstrated that an active lifestyle and an adequate fitness level may attenuate these adverse effects (17). In addition, genetic and early programming features have been associated with low-grade inflammation in young people (18). However, to date, the potential anti-inflammatory effects of fitness in children are not entirely clear. The results obtained in the present work add evidence of the positive effect of an adequate fitness status during childhood related with inflammatory status. So, those participants with HF levels, showed also lower BMI and WC, suggesting that fitness and gender might influence adipose tissue metabolism programming changing these biomarkers in this early stage of life, before puberty changes.

The association between fitness and fibrinolytic activity has been scarcely studied in prepubertal children, showing a high disparity among the results obtained in studies conducted on both variables. It has been described a strong correlation between fitness and PAI-1 levels in children after adjustment for age and fat mass (19) even though not all studies have observed this association (1). Most of these studies have been carried out on children with obesity. Although authors adjusted by fat mass, elevated adiposity might be associated with a higher physiological deposit of PAI-1 that might explain these differences. Here, although the participants were normal-weight, those in the LF group showed higher levels of PAI-1, similarly as reported Barbeau et al. (20) under the same conditions. Our differences might be due to a stricter criterion to classify the participants as high or low fitness levels. However, it seems to be an indirect effect, where an increase in the level of fitness would give as result a decrease in the BMI and consequently a reduction in PAI-1 plasma levels. Moreover, in the present study, PAI-1 was positively associated with other biomarkers such as resistin, leptin, IL-8, HGF, and TNF- α, and with WC (Table 4). Decreased resistin concentrations during fasting have been associated with a decrease in the percentage of body fat in adolescents (21). Therefore, an adequate fitness level may have an indirect effect on the inflammation status.

Leptin has been reported to be independently and inversely related to fitness (7). There is scarce literature regarding the association between fitness and leptin in prepubertal children. The current findings suggest that low levels of aerobic fitness, as well as elevated serum leptin, are major risk factors for the clustering of metabolic risk factors in obese (22) or those presenting a medium-healthy or unhealthy status (23). Our results support these findings showing higher levels of leptin in LF group even being younger children. On the other hand, fitness might stimulate insulin sensitivity and induce a decrease in insulin release, which in turn might reduce leptin levels. A reduction in levels of obesity seems to produce a reduction on TNF-α, which lead to a decline in leptin and an increase in adiponectin and insulin sensitivity (24). Therefore, it appears that the action of adiponectin depends on changes in fat mass (25). According to the previous hypothesis, our results might suggest an influence of fitness on leptin in healthy children and possibly on TNF-α, but not on adiponectin. In fact, this study supports the relationships among TNF-α with leptin, adiponectin and BMI (that includes fat mass).

The NGF is a well-known regulator of differentiation, plasticity, and phenotype of sensory and sympathetic neurons during the entire lifespan (26). Alterations in NGF tissue concentrations lead to altering nutritional factors in the muscle fibers after high-intensity exercises (27). Moreover, it has been demonstrated that inflammation can enhance the synthesis of NGF in the tissues (28) and give rise to an increase in other markers such as TNF-α. Therefore, the present study shows that the HF group could have a lower risk of inflammatory events and NGF-derived effects.

Parallel, the relationship between gender and inflammation biomarkers has not been clarified. According to some authors, girls have higher levels of leptin and resistin and lower levels of TNF-α in comparison with boys (1). As regards adiponectin levels in young people, it has been reported that prepubertal girls present higher adiponectin concentrations than boys (1, 9). It has been suggested that the effects of age on adiponectin levels in girls could be explained by their BMI and total fat mass (29). However, it is still difficult to explain the differences in inflammatory markers by sex, mainly in children because the changes among genders have usually been associated with their hormonal status. Therefore, considering our results, it seems that girls are low protected against inflammation during the prepubertal stage, although it might change once puberty is developed.

Finally, when we observed the effect of fitness levels on both gender separately, the girls with an elevated fitness were shown to be protected against the development of an inflammatory process by presenting higher levels of adiponectin and lower plasma levels of leptin and NGF.

As limitations of the present study, it is important to emphasize the high difficulty to detect inflammatory molecules in the blood samples due to the low concentrations in children without pathology. Moreover, this study has carried out with a sample from a city in Spain, so it will be interesting to expand to other regions and different stages of life.

## Conclusions, Future Scope, and Limitations

This study contributes to add new information about the effect of fitness and gender on plasma adipokines and inflammatory cytokines in healthy prepubertal children. An adequate or higher fitness level seems to be a protective factor against the development of obesity, metabolic risk and inflammation, decreasing leptin and NGF levels, so as contributing to control adiposity at the prepubertal stage. However, research focus in this and other stages of life should be developed to generate more accurate knowledge and relationships between a low grade of inflammation and cardiorespiratory fitness.

## Data Availability Statement

The raw data supporting the conclusions of this article will be made available by the authors, without undue reservation.

## Ethics Statement

The studies involving human participants were reviewed and approved by the Institutional Ethics Committee Reina Sofia University Hospital, Cordoba, Spain. Written informed consent to participate in this study was provided by the participants' legal guardian/next of kin.

## Author Contributions

FL-C: conceptualization, data curation, formal analysis, methodology, writing—original draft, and review and editing. CA: conceptualization, funding acquisition, investigation, and writing—review and editing. JP-N and JB-S: conceptualization, methodology, and review and editing. AG: conceptualization, methodology, and writing—original draft. MG-C: conceptualization, investigation, methodology, project administration, resources, writing—original draft, and review and editing. All authors contributed to the article and approved the submitted version.

## Funding

This work was supported by the Premio Salud Investiga, Junta de Andalucía, Spain and CIBEROBN.

## Conflict of Interest

The authors declare that the research was conducted in the absence of any commercial or financial relationships that could be construed as a potential conflict of interest. The handling editor JD-C declared a shared affiliation with the authors CA and AG at the time of review.

## Publisher's Note

All claims expressed in this article are solely those of the authors and do not necessarily represent those of their affiliated organizations, or those of the publisher, the editors and the reviewers. Any product that may be evaluated in this article, or claim that may be made by its manufacturer, is not guaranteed or endorsed by the publisher.
